# Highlight: Nonmammalian Transcription Factors Make Mice Mothers Less Loving

**DOI:** 10.1093/gbe/evu088

**Published:** 2014-05-19

**Authors:** Danielle Venton

For the first time, a complex behavior, mothers caring for their offspring, has been linked to a semimysterious repeating amino acid sequence common to mammals. These repeats, found more often in mammals than other vertebrates, have been seen in the past to affect protein–protein interactions, transcriptional regulation, and phenotypic variation.

Japanese biologists Shintaroh Ueda, Den'etsu Sutoo, and colleagues from the University of Tokyo and the University of Tsukuba wanted to know whether these repeats had evolutionary significance. To investigate, the researchers replaced a gene in mice with its ortholog from western clawed frogs. The amphibian version of the transcription factor Pou3f2 is repeat-free, whereas the mammalian version is rich in glycine, glutamine, and proline repeats. They report their findings in a recent issue of *Genome Biology Evolution* (Makoto et al. 2014). Before starting the experiments, says Ueda, it was hard to guess what changes they would see, but he felt confident something would appear.

The effects were dramatic: Carrying nonmammalian *Pou3f2* essentially turned female mice into bad mothers. Though giving birth to healthy, normal-sized pups, a dam was less interested in her pups, and less likely to fetch them and bring them to her nest. Most pups born to “knock-in” mothers did not survive to weaning, whereas most pups born to normal mothers did.

“This is significant, I think, for reminding us that we shouldn't just be looking at the regions of proteins traditionally considered functional,” says Noel Faux, a research fellow at the Florey Institute for Neuroscience and Mental Health in Melbourne, Australia, who was not involved in the research. “Regions we wouldn't normally consider obviously do have a function here.”

Without the repeating homopolymeric amino acids, the mice did not have as much dopamine and serotonin in their brains—past studies have shown that these two neurotransmitters heavily affect how mammalian mothers care for their offspring. Dopamine and serotonin regulate many other complex thoughts and behaviors: Anxiety, fear, mood control, motivation, cognition, reward, movement, regulation of body temperature, and drug abuse.

“We hypothesize that the regulation of these functions is not sufficiently strict in nonmammals including amphibians,” write Ueda et al. Although animals such as fish and amphibians behave more or less instinctively, mammals behave in ways that are not necessarily instinctive, they show emotion, bond socially, and nurturing their young.

“This study opens a host of interesting questions,” says Faux. “If you give these [knock-in] mice tests for memory, anxiety or depression, how do they perform?”

In a more technical vein, he is curious to know how the structure of transcription factor Pou3f2 differs between knock in and normal mice. Although orthologous proteins fold in a similar manner, the repeats presumably, he says, affect structure in some way. Does the length of the repeats affect neurotransmitter levels and behavior, he wonders. Also, in this study, all three repeat-rich regions of the mammalian *Pou3f2* gene were swapped for no-repeat amphibian versions. He would like to see experiments removing each of those regions individually, to see which has the strongest effect on behavior.

Faux says many of his colleagues will be intrigued by the study. It may, he hopes, bolster support for their research: “The more evidence that these [repeats] do have an impact the more the granting bodies are likely to take it on board. It's sort of a new area and people are still a bit skeptical that these things can have an effect on fairly large behaviors.”

Ueda also believes that this field of research is just beginning. There are hundreds of mammalian genes with homopolymeric amino acid repeats, he says, and the majority are evolutionarily conserved in length across mammals and have important roles in transcription, translation, and signaling processes. These knock-in mice, says Ueda, are available as models for researchers to deeply dive into the characteristics that make mammals unique.
